# Fatal Steam Pop Complicating Investigational SESAME Transcatheter Myotomy Using Off-the-Shelf Equipment

**DOI:** 10.1161/CIRCINTERVENTIONS.125.016252

**Published:** 2026-01-26

**Authors:** Robert J. Lederman, Adam B. Greenbaum, Rim N. Halaby, Annette M. Stine, Jaffar M. Khan, Christopher G. Bruce, Toby Rogers, Patrick T. Gleason, Ozlem Bilen, Vasilis C. Babaliaros

**Affiliations:** Division of Intramural Research, National Heart, Lung, and Blood Institute, National Institutes of Health, Bethesda, MD (R.J.L., R.N.H., A.M.S., J.M.K., C.G.B., T.R.).; Emory University, Atlanta, GA (A.B.G., P.T.G., O.B., V.C.B.).; St Francis Hospital and Heart Center, Roslyn, NY (J.M.K.).; MedStar Washington Hospital Center, Georgetown University, Washington, DC (T.R.).

**Keywords:** cardiac catheterization, cardiomyopathy, hypertrophic, electrosurgery, heart failure, heart septal defects, myotomy, ventricular outflow obstruction

Transcatheter myotomy (Septal Scoring Along Midline Endocardium [SESAME]) has entered clinical practice^1–3^ as an alternative to surgical myectomy and transcatheter alcohol septal ablation for obstructive hypertrophic cardiomyopathy. The myotomy tends to widen over weeks without tissue removal, and incident heart block appears low.^1^

We prospectively evaluated SESAME using commercial off-the-shelf devices (URL: https://www.clinicaltrials.gov; Unique identifier: NCT06269640) under Food and Drug Administration Investigational Device Exemption license (G220158) and National Institutes of Health institutional research board oversight, to characterize physiology and clinical outcomes. Key selection criteria included consenting adults with symptomatic (New York Heart Association Class ≥III) hypertrophic cardiomyopathy and left ventricular outflow obstruction; septal diastolic thickness ≥12 mm; predicted residual laceration thickness ≥6 mm; multidisciplinary heart team determination of high surgical risk and suitability for SESAME; no prior septal reduction therapy; and informed patient preference for SESAME over cardiac myosin inhibitors and transcatheter alcohol septal ablation.

## Patient

An 86-year-old woman with obstructive hypertrophic cardiomyopathy had New York Heart Association III symptoms and recurrent heart failure admissions. Medications included verapamil and carvedilol; she declined mavacamten. Echocardiography showed left ventricular ejection fraction 0.75, resting left ventricular outflow tract gradient 37 mm Hg, and 130 mm Hg after Valsalva. Cardiac magnetic resonance (1.5T) showed no late gadolinium enhancement but elevated native T1 (1094±71 ms) and extracellular volume (30.8%). She was considered an excessive risk for surgical myectomy. After counseling about the novelty, limited global experience, and risks including ventricular septal defect and death, she chose SESAME instead of transcatheter alcohol septal ablation and consented to participate in the protocol.

## SESAME Procedure

**Figure 1.**
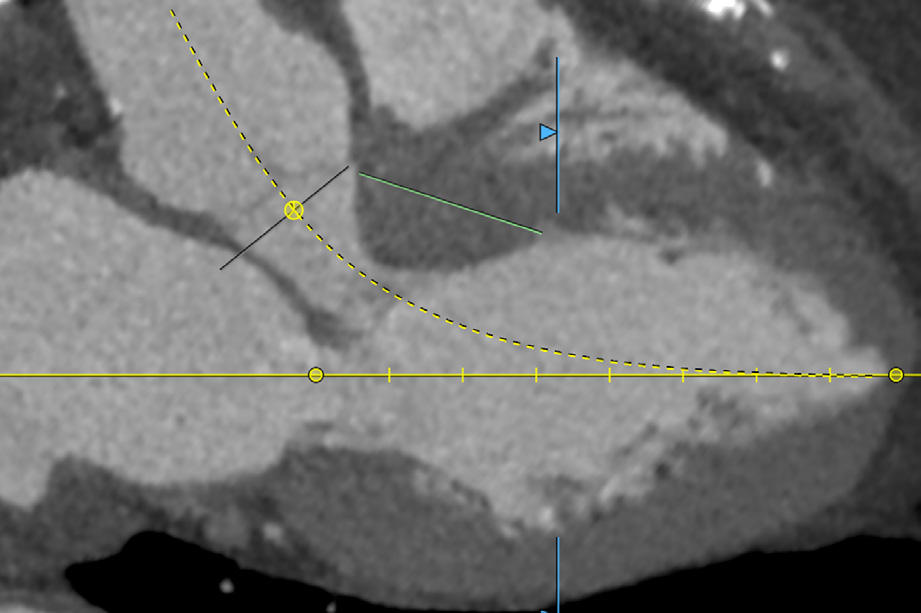
The Septal Scoring Along Midline Endocardium trajectory (green line) is planned on computed tomography to shave the hump and to enter the septum immediately below the aortic valve.

**Figure 2.**
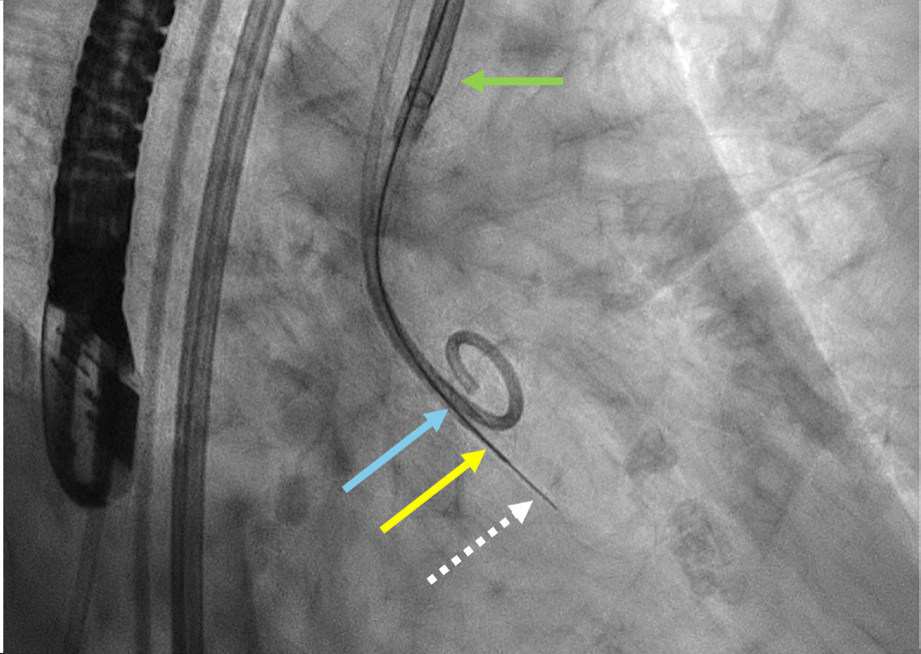
**Fluoroscopy depicts initial guidewire navigation through the base of the ventricular septum.** A pigtail catheter marks the aortic root. A deflectable guiding sheath (green) positions the guiding catheter (blue arrow) to direct a 0.014″ guidewire (dotted white arrow) inside a 0.014″ microcatheter (yellow).

**Figure 3.**
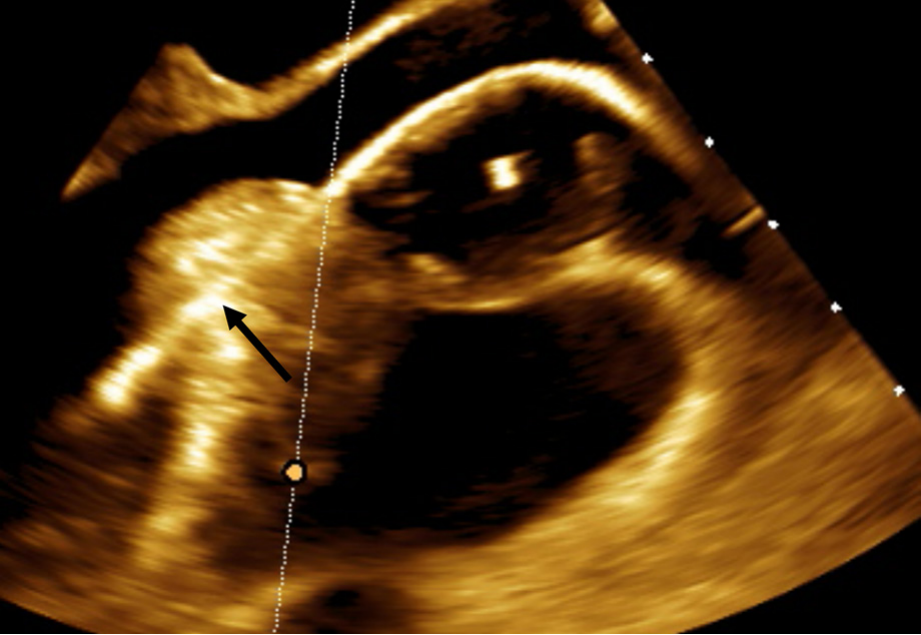
The intramyocardial course of the coaxial guidewire and microcatheter (arrow) is visualized by transesophageal echocardiography before laceration.

**Figure 4.**
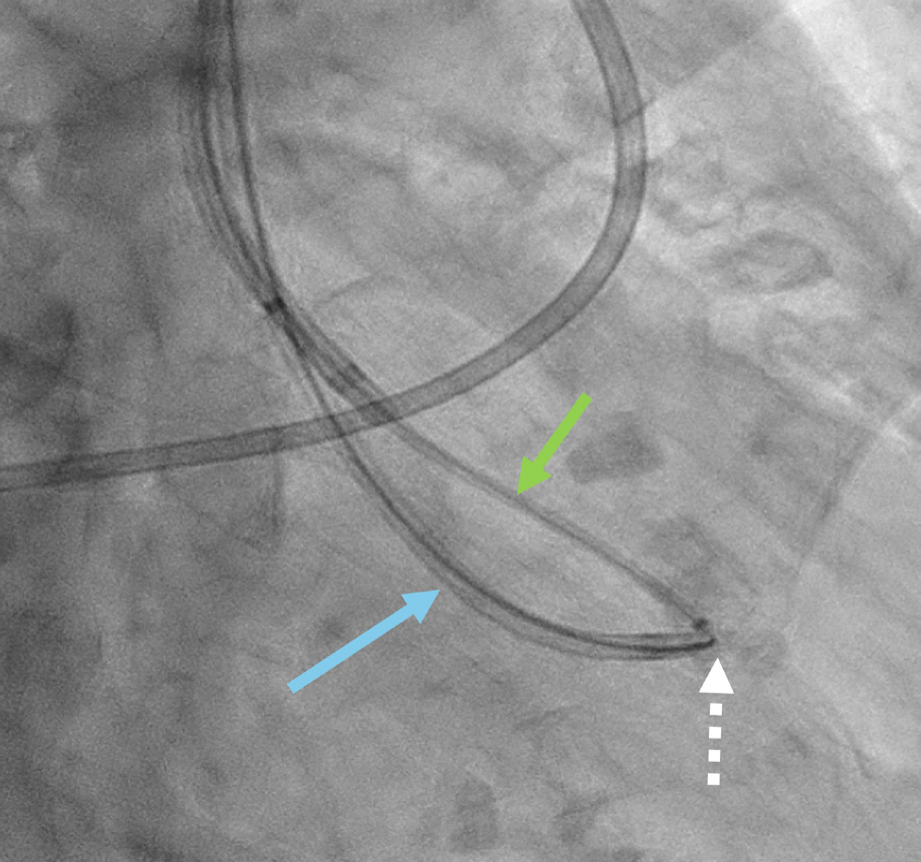
**This figure depicts the catheter configuration on initiating the Septal Scoring Along Midline Endocardium electrosurgical laceration.** The intramyocardial limb of the guidewire is insulated with a 0.035″ microcatheter (green arrow) that allows cooling and venting saline flush to be administered during energy application. The intracavitary limb of the guidewire is covered with a 0.014″ insulating microcatheter (blue arrow). The flying-V is the only exposed electrosurgery surface (dotted white arrow).

The procedure was planned on computed tomography (Figure 1). Under general anesthesia and with transesophageal echocardiography guidance, the basal LV septum was engaged with a transfemoral deflectable guiding sheath (Agilis; Abbott) and a long-tip guiding catheter (Champ 1.5; Medtronic). A 0.014″ guidewire (AstatoXS40; Asahi) via a 0.014″ microcatheter (CorsairProXS; Asahi) entered across the endocardium after a brief electrosurgical pulse (Figure 2) and advanced through the myocardium 8 mm below the septal surface as confirmed by multiple transesophageal echocardiography views (Figure 3). The guidewire was ensnared and externalized through a second retrograde guiding catheter. A standard flying-V laceration surface was created by denuding the insulation from the innersurface of a midshaft kink in the intramyocardial AstatoXS40 guidewire. The intramyocardial limb was covered with a 0.035″ microcatheter (Navicross) for insulation and irrigation during electrosurgery. The intracavitary limb was insulated with the repositioned 0.014″ microcatheter. SESAME myotomy was performed using 50 W electrosurgery while traction was applied to both guiding catheter limbs and while saline was injected into the intramyocardial catheter limb (Figure 4). Electrosurgery energy was applied in multiple bursts lasting up to 3 seconds. The left ventricular outflow tract gradient fell to 10 mm Hg at rest and 40 mm Hg after provocation.

**Figure 5.**
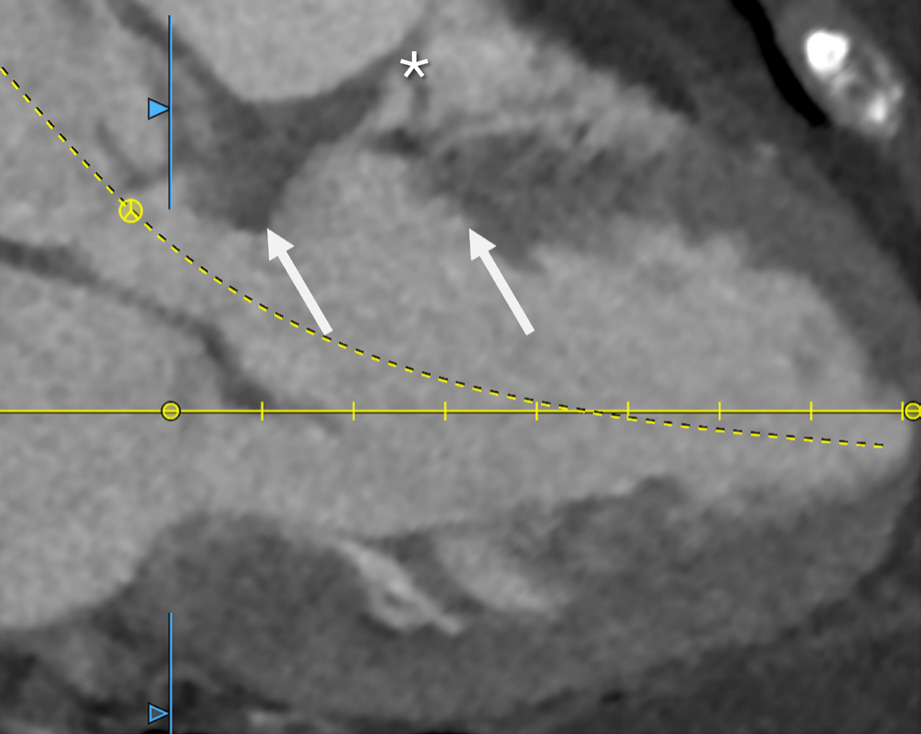
**Computed tomography after Septal Scoring Along Midline Endocardium shows the lengthwise extent (white arrows) of the myotomy, along with a ventricular septal defect (white asterisk).** The complex ventricular septal defect pattern is more consistent with steam pop than with excessively deep myotomy.

**Figure 6.**
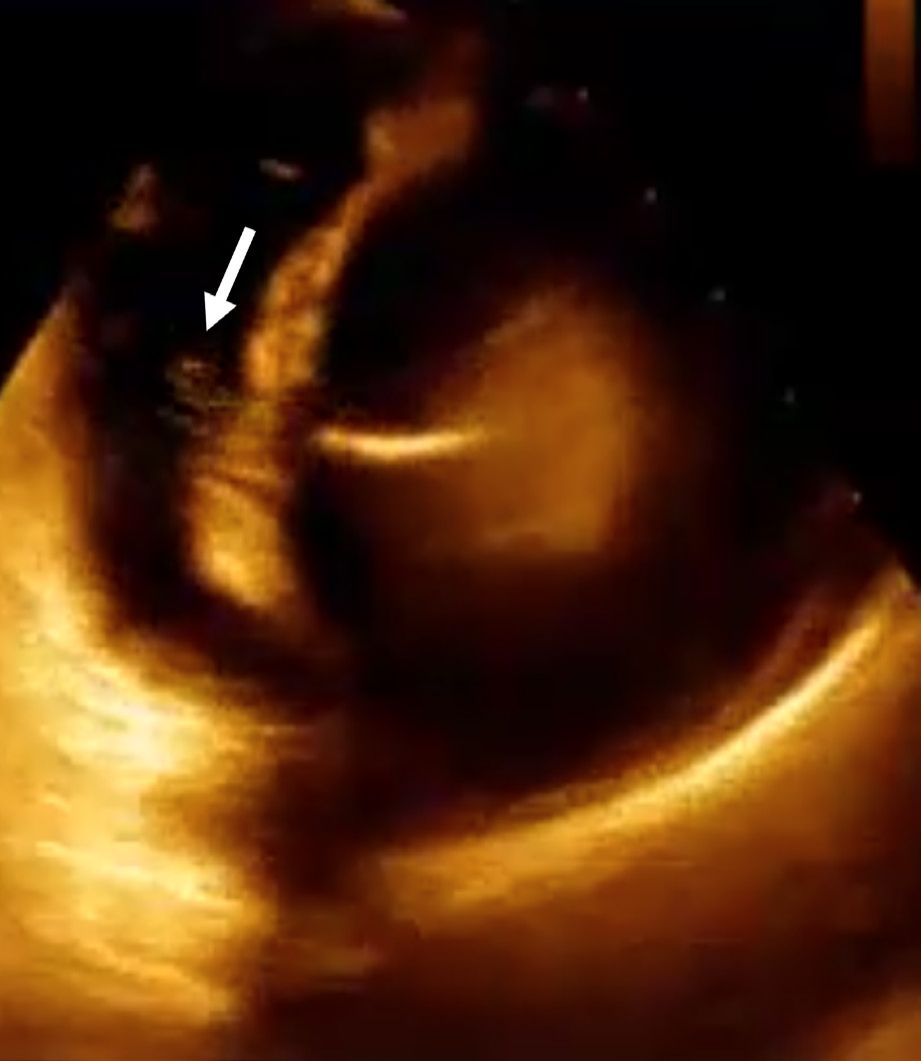
Steam-pop bubbles (arrow) were released into the right ventricle during electrosurgery, as found on transesophageal echocardiography.

Excessively deep laceration was evident out of proportion to the intramyocardial guidewire position on echo and computed tomography (Figure 5) with a restrictive ventricular septal defect. In retrospect, transesophageal echocardiography showed RVOT steam-pop bubbles during electrosurgery (Figure 6). Afterwards, she developed progressive shock, sustained cardiac arrest, and died on hospital day 3 during attempted emergency salvage percutaneous ventricular septal defect closure.

**Figure 7.**
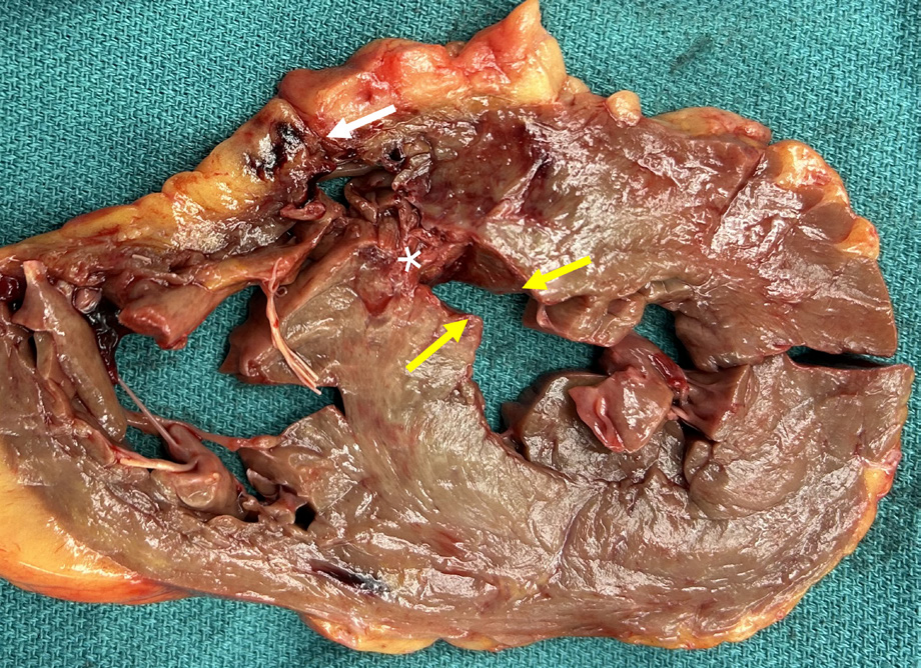
**Postmortem Septal Scoring Along Midline Endocardium (SESAME) shows splayed myotomy (yellow arrows).** The ventricular septal defect (white asterisk) exhibits a jagged morphology suggesting steam pop. The attempted emergency salvage ventricular septal defect repair caused additional right ventricular wall injury (white arrow) not directly related to SESAME.

Postmortem examination of the heart demonstrated the intended SESAME longitudinal myotomy plus a deeper jagged tract communicating with the RV along the moderator band (Figure 7). The jagged tract morphology suggested uncontrolled intramyocardial vapor release (steam pop) as the likely mechanism.

## Discussion

Steam pop describes an explosive water vapor release that tunnels unpredictably within the myocardium during ablation, typically during radiofrequency ablation of ventricular arrhythmia.^4^ The SESAME technique tested in this protocol relies on tissue vaporization from the flying-V, which requires extremely high temperatures, and which requires accumulated gas to vent freely into the left ventricular chamber.

We hypothesize that deep, inadequately vented intramyocardial heating of the commercial off-the-shelf guidewire system created a steam pop with transmural extension. Another possibility is that the intramyocardial wire position was deeper than recognized.

Patients and clinicians should weigh the risk of steam pop carefully when considering SESAME using off-label tools. In the meantime, SESAME operators should attempt to limit deep heating by modulating electrosurgery energy, insulation, venting, and irrigation. For a given power input and electrode configuration, temperature is related to the duration of energy application and to countervailing heat dissipation. Multiple short applications of energy (such as <1 second) might reduce maximum deep tissue temperature, leading to steam accumulation. Purpose-built SESAME catheter systems should be designed to limit deep intramyocardial heating.

## Article Information

### Sources of Funding

This research was supported by the Intramural Research Program of the NIH, Z01-HL006040 (to Dr Lederman). The contributions of the NIH authors are considered Works of the United States Government. The findings and conclusions presented in this paper are those of the authors and do not necessarily reflect the views of the NIH or the US Department of Health and Human Services.

### Disclosures

Drs Lederman and Bruce are coinventors on applicable patents, assigned to National Institutes of Health (NIH). Dr Greenbaum reports consulting for Abbott Vascular, Edwards Lifesciences, Excision Medical, and Medtronic; equity interest from Transmural Systems and Excision Medical. Institutional research support from Abbott Vascular, Ancora Heart, Edwards Lifesciences, Gore Medical, JenaValve, Medtronic, Polares Medical, Transmural Systems, and 4C Medical. Dr Khan reports consulting/proctoring fees from Abbott, Edwards Lifesciences, and Medtronic; equity interest from Transmural Systems and Cuspa Medical; intellectual property: coinventor on patents, assigned to the NIH, on leaflet modification devices. Dr Rogers reports consulting for Edwards Lifesciences, Medtronic, Boston Scientific, Abbott, Anteris, and Transmural Systems; advisory board for Medtronic, Boston Scientific; equity interest for Transmural Systems; and intellectual property: coinventor on patents, assigned to NIH, on applicable patents. Dr Gleason reports institutional research contracts for clinical investigation of transcatheter aortic, mitral, and tricuspid devices from Edwards Lifesciences, Abbott Vascular, Medtronic, and Boston Scientific. Dr Bilen reports consulting and speaking for Bristol Myers Squibb and Cytokinetics. Dr Babaliaros reports consulting for Abbott Vascular, Edwards Lifesciences, and Medtronic; equity interest from Transmural Systems; and Institutional research support from Abbott Vascular, Ancora Heart, Edwards Lifesciences, Gore Medical, JenaValve, Medtronic, Polares Medical, Transmural Systems, and 4C Medical. The other authors report no conflicts.
